# Multifocal gastric gastrointestinal stromal tumors (GISTs) with lymph node metastases in children and young adults: A comparative clinical and histomorphological study of three cases including a new case of Carney triad

**DOI:** 10.1186/1746-1596-6-52

**Published:** 2011-06-10

**Authors:** Claudia Otto, Abbas Agaimy, Alexander Braun, Jochen Rädecke, Jens Hoeppner, Gerald Illerhaus, Martin Werner, Udo Kontny, Florian Haller

**Affiliations:** 1 Institute of Pathology, University Hospital Freiburg, Germany; 2 Institute of Pathology, University Hospital Erlangen, Germany; 3 Division of Pediatric Hematology and Oncology, Center for Pediatrics and Adolescent Medicine, University Medical Center, Freiburg, Germany; 4 Department of Gastroenterology, University Hospital Freiburg, Germany; 5 Department of General Surgery, University Hospital Freiburg, Germany

## Abstract

**Background:**

Gastrointestinal stromal tumors (GISTs) are the most frequent mesenchymal tumors of the gastrointestinal tract usually occurring in the 6^th ^to 7^th ^decade of life, while their occurrence in children is rare (1-2%).

Carney triad (CT), a non-hereditary association of gastric GIST with pulmonary chondroma and/or extraadrenal paraganglioma, is an even much rarer disease (to date ~120 cases reported worldwide) usually affecting young adult females. Pediatric GISTs differ from CT-associated GISTs solely by the absence of other components of the triad and are completely different from sporadic GISTs of the adult. Both, pediatric and CT-GISTs, metastasize frequently to regional lymph nodes (29%) and are usually wild type (WT) for common KIT-/PDGFRA mutations.

**Case presentation and results:**

We compare one new CT GIST with two pediatric/young adult multifocal gastric GISTs presenting with lymph node metastases. We put special focus on histomorphological growth pattern in the primary tumors and in the metastases.

The two cases of pediatric/young adult GIST without the other components of CT showed all the features of the triad: female gender, young age, multifocal antral-based gastric GIST with biphasic histological growth pattern, lymph node metastases, hypercellularity and WT status for common KIT-, PDGFRA- and B-RAF mutations.

**Discussion and conclusion:**

Pediatric/CT-associated GISTs and sporadic GISTs of the adults differ significantly from each other with regard to patients' age, gender, tumor localisation, histomorphological growth pattern, mutational status and risk for metastasis. Our cases of pediatric/young adult GISTs show all characteristics of CT except for the absence of other components of the triad.

Therefore these GISTs are probably not sporadic, but may represent either early manifestation or *forme fruste *of the CT. Thus, these patients need to be regularly examined for the development of extraadrenal paraganglioma or pulmonary chondroma.

## Background

Gastrointestinal stromal tumors (GISTs), the most common mesenchymal neoplasms of the gastrointestinal tract (app. 70%) [1-4] usually affect adults in the 6^th ^and 7^th ^decade of life without any proven gender prevalence [5-8]. However, GISTs represent only a small fraction of all gastrointestinal tumor entities seen in adults (≤ 2%) [6-8] and they are rare in childhood and adolescence (1-2% of all GIST cases) [5,9,10]. GISTs in childhood/adolescence can occur as sporadic disease unrelated to a syndrome, present as a familial disorder (e.g. Carney-Stratakis syndrome) or be a part of the non-hereditary Carney triad (CT). On the other hand, GISTs in patients affected by neurofibromatosis type 1 usually present at a later age (mean age at presentation = 46 years) [11].

In 1977 J. Aidan Carney first described the association of gastric epithelioid leiomyosarcoma (later renamed as gastrointestinal stromal tumor) with pulmonary chondroma and functioning extraadrenal paraganglioma of unknown origin, which is today known as CT [12,13].

CT is rare with approximately 120 published cases worldwide to date, usually affects females (88%) in their 2^nd ^and 3^rd ^decades [14,15] and often presents with unpredictable outcome [15]. For the diagnosis of CT at least two of the three major components are necessary. Seventy three percent of the patients present with incomplete CT characterized by manifestation of two components of the disorder [14,15]. The most common combination is the association of GIST and pulmonary chondroma (35.6%) [15]. Recently two other possibly associated tumors were added: leiomyoma of the esophagus and adrenal cortical adenoma [15,16]. The gastric GISTs in CT are usually multifocal, antral based and show a wild type (WT) for common mutations in receptor tyrosine kinase gene KIT and for homologue oncogene platelet-derived growth factor receptor α gene (PDGFRA) [17,18] and they present with typical biphasic growth pattern [15]. Approximately 29% of the patients develop regional lymph node metastases [15,19,20] contrasting with the rarity of lymph node metastasis in sporadic GISTs in adults (≤ 2%) [19-22]. Therefore lymph node dissection is not recommended in adult GIST patients [2,20,23]. Despite the high tendency for metastasis, especially to regional lymph nodes (29%) and liver (ca. 25%), the clinical course of the CT-GISTs is usually indolent with long survivals even with metastatic disease [15,16].

## Case presentation and results

In this study, we describe two new cases of multifocal gastric GIST with lymph node metastases in pediatric/young adult females and compare the findings with that of a new CT-GIST with special focus on histomorphological growth pattern, mutational status and the pattern of metastasis.

### Case 1

The first patient, a 15-year-old girl was admitted to the hospital with anaemia caused by upper gastrointestinal bleeding. An antral-based multifocal gastric tumor, suspicious for GIST, was detected in the greater curvature of the stomach. The largest tumor nodule measured 7 cm in diameter and showed mucosal ulceration. Furthermore multiple liver metastases (maximum diameter: 4 cm), were detected preferentially in the left lobe. After confirmation of the diagnosis GIST by open biopsy the patient underwent therapy with imatinib (400 mg once a day × 9 weeks followed by 2 × 400 mg/d × 2 weeks). No change in tumor size was observed and the patient underwent gastrectomy and regional lymphadenectomy. The patient has been since then (for now 6 years) on different tyrosine kinase inhibitors including motesanib, sunitinib and nilotinib with only slight progression in the size of liver metastases [24].

The primary tumor showed biphasic histomorphological growth pattern with both, spindled and epithelioid cells. Additionally, hypercellularity as well as plasmacytoid character of the tumor cells was seen. Mitotic count was 5/50 high power fields (HPF; 50 HPFs corresponded to 10 mm^2^). In the liver metastases the tumor cells demonstrated a paraganglioma-like growth pattern with small compact nests, whorls and "Zellballen"-like aggregates of epithelioid tumor cells. Furthermore, histological examination of a 2.5 × 1.8 × 1.2 cm intrathoracic paraaortal nodule thought to represent lymph node metastasis revealed a paraganglioma leading to the diagnosis of incomplete CT with synchronous gastric GIST and extraadrenal paraganglioma.

**Figure 1:**
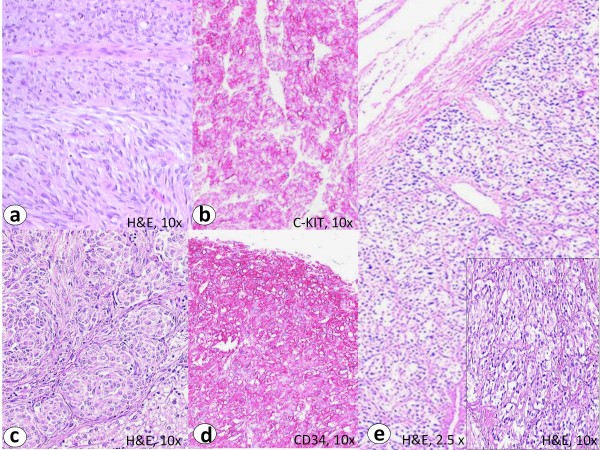
**The primary tumor of the CT-GIST (case 1) showed a biphasic histomorphological growth pattern with spindled and epithelioid cells (a, H&E staining)**. Immunohistochemistry revealed an intensive predominantly membranous staining with C-KIT (b) and CD34 (d). The liver metastases revealed small nests and "Zellballen" of tumor cells with epithelioid growth pattern (c, H&E staining). The paraganglioma showed a characteristic nested pattern (e, H&E staining). Note similarity to hepatic metastasis from GIST in c.

Immunohistochemistry of the primary tumor showed intensive membranous staining for CD117 and CD34 (figure 1).

Mutational analysis showed a WT for KIT (exons 9, 11, 13, 17) and PDGFRA (exons 12, 14, 18) as well as for B-RAF exon 15 V600E mutations. However, further examination disclosed a rare PDGFRA exon 10 polymorphism (c. 1432 T > C; p. S478P) that is present on the DNA level but does not lead to alterations on the protein level.

### Case 2

The second patient, a 13-year-old girl presented with long-lasting anaemia caused by upper gastrointestinal bleeding and worsening general condition. As the initial therapy with ferrous sulphate showed no benefit, ultrasound of the abdomen and magnetic resonance tomography were performed. During these examinations, an 8.1 × 7.1 × 5.2 cm bulky mass attached to the lesser curvature of the stomach as well as multiple hyperdense areas in both lobes of the liver (max. 2.2 cm) were discovered. Subsequent gastric endoscopy revealed an exophytic bleeding multilobular gastric tumor with mucosal ulceration. By positron emission tomography the hyperdense areas in the liver were confirmed as metastases of the gastric tumor. The patient then underwent neoadjuvant imatinib therapy followed by 4/5 resection of the stomach with gastrojejunostomy and regional lymphadenectomy of the lesser curvature. Because of the multiplicity of the liver metastases surgeons decided against resection. The patient has been since then for 2 years on imatinib (400 mg/d) with no major change in size of liver metastases [24].

**Figure 2:**
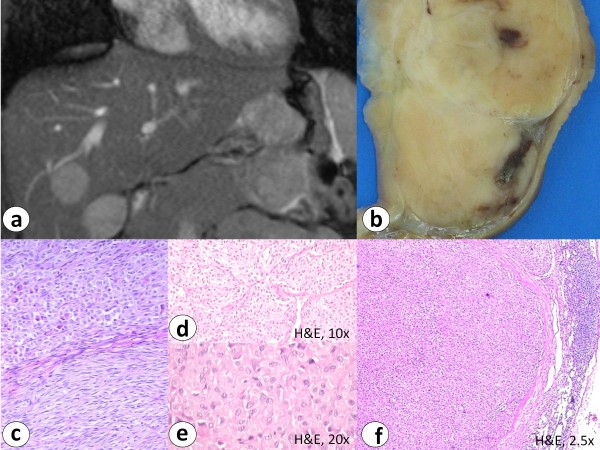
**Magnetic resonance tomography in case 2 showed an exophytic, lobulated and ulcerated antral mass diagnosed as gastric GIST with multiple smaller satellite tumor nodules, liver (a, b) and lymph node metastases (f) (H&E staining)**. Histology reveals biphasic histomorphological growth pattern (c) with fibromuscular septa (d) and hypercellularity (e) (H&E staining).

Histological examination of the gastric specimen revealed an 8 cm multifocal gastric GIST with mitotic count of 4/50 HPF (intermediate risk after Fletcher et al. and low risk after Miettinen et al.). Furthermore multiple regional lymph node metastases with epithelioid growth pattern were verified. The primary tumor showed biphasic histological growth pattern. Immunohistochemistry showed distinct cytoplasmic and membranous expression of CD117 and CD34 (figure 2).

The tumor was WT for KIT exons 9, 11, 13 and 17, PDGFRA exons 10, 12, 14 and 18 as well as for B-RAF exon 15 mutations.

### Case 3

The third patient, a 40-year-old woman currently presented with liver and lymph node metastases (celiac trunk).

Exploration of her medical history uncovered that the patient underwent partial gastrectomy 17 years ago (23 years old at that time) because of a gastric mesenchymal tumor with hepatic and peritoneal metastasis. At that time the tumor was classified as gastric leiomyosarcoma or malignant stromal tumor of the stomach and the patient was treated with high-dose chemotherapy and autologous stem-cell transplantation [25]. leading to 16 years long term remission. The patient reported nonspecific upper gastrointestinal complaints since the age of 13.

**Figure 3:**
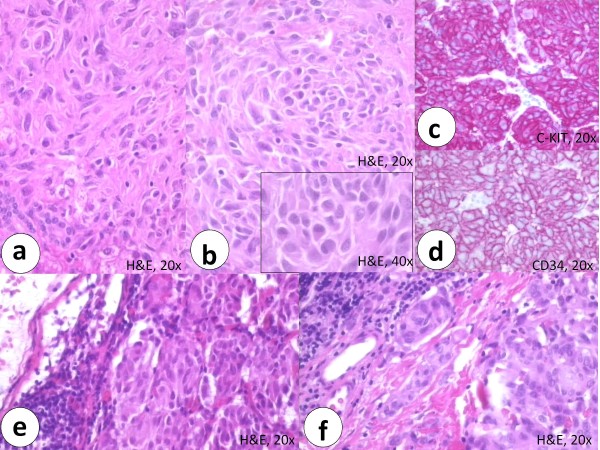
**The histomorphological growth pattern of the liver metastasis in case 3 showed a hypercellular tumor (a) with plasmacytoid cells (b and inset) (H&E staining)**. Immunohistochemistry of the liver metastasis showed intensive expression of predominantly membranous C-KIT (c) and distinct membranous CD34 (d) staining. The lymph node metastases revealed closely packed hypercellular tumor nodules (e) with lymphangioinvasion (f) (H&E staining).

Unfortunately slides or paraffin blocks of the primary gastric tumor were not available any more for revision, but the histological description found in the medical report was consistent with a GIST. The patients young age, female gender, tumor localisation, mutational status (WT for KIT, PDGFRA and B-RAF mutations), (histo)morphological growth pattern (multilobular gastric tumor, biphasic growth pattern, hypercellularity, plasmacytoid cell morphology), immunohistochemistry findings and the pattern of metastasis (lymph node and liver metastases) are consistent with the previous two cases (figure 3). Up to now there is no evidence for pulmonary chondroma or extraadrenal paraganglioma in this patient and she is well.

## Discussion and conclusion

Sporadic GISTs, usually affecting adults in the 6^th ^and 7^th ^decade of life [5-8], are rare in childhood and adolescence [5,9,10]. Currently, the management of sporadic pediatric/young adult GISTs is not standardized, probably due to the relatively low number of reported cases (113 published cases below the age of 21) [26] and lack of complete understanding of the molecular pathogenesis of these usually WT-GISTs.

However, current understanding suggests that sporadic pediatric GISTs represent a tumor entity distinct from common GISTs in adults but with striking similarity to CT-GISTs.

WT pediatric GISTs are distinguished from CT-GISTs merely by the absence of other triad components in the latter. In contrast to most of the sporadic GISTs of the adults, both CT-GISTs and pediatric GISTs are WT for common mutations of the receptor tyrosine kinase genes KIT and PDGFRA [17,18]. Pediatric and CT-GISTs present commonly in childhood and adolescence, show a strong female predilection (88%), are mostly antral-based, multifocal, show a distinct biphasic histomorphological growth pattern and frequently metastasize to regional lymph nodes, thus strikingly contrasting with common GISTs seen in adults (≤ 2% lymph node metastases). The reason for this high rate of lymph node metastasis is not known [20].

Similarly, our two cases of pediatric/young adult GIST without other components of CT (case 2 and 3) showed all the features of the CT-GISTs: young age, female gender, antral-based gastric GIST, multifocal tumor growth, biphasic histological growth pattern, hypercellularity, WT status for common KIT-, PDGFRA- and B-RAF mutations and presence of lymph node metastases (table 1).

**Table 1:** Comparison between Carney triad GIST (case 1), pediatric (case 2) and young adult GIST (case 3)

	CT-GIST (case 1)	Pediatric GIST (case 2)	Young adult GIST (case 3)
**Age at diagnosis (years)**	15	13	23

**Gender**	female	female	female

**Tumor localisation**	stomach, greater curvature, antrum	stomach, lesser curvature, antrum	stomach, corpus

**Growth pattern**	multinodular	multinodular	multinodular

**Metastases**	liver (lymphangioinvasion)	liver lymph nodes	liver lymph nodes

**Histomorphology**	biphasic (spindled and epithelioid), hypercellular, plasmacytoid aspect	biphasic (spindled and epithelioid), hypercellular, plasmacytoid aspect	liver metastasis: hypercellular, plasmacytoid aspect lymph node metastasis: closely packed hypercellular tumor nodules with lymphangioinvasion

**Immunohisto-** **chemistry**	C-KIT/CD34 + (predominantly membranous)	C-KIT/CD34 + (predominantly membranous)	C-KIT/CD34 + (predominantly membranous)

**Mutational status**	WT for common C-KIT (exon 9,11,13,17) and PDGFRA (exon 12,14,18) mutations PDGFRA Exon 10 polymorphism (c. 1432 T > C; p. S478P, no alteration on protein level)	WT for common C-KIT (exon 9,11,13,17) and PDGFRA (exon 12,14,18) mutations	WT for common C-KIT (exon 9,11,13,17) and PDGFRA (exon 12,14,18) mutations

**Associated diseases**	paraaortal paraganglioma	?	?

CT = Carney triad, GIST = gastrointestinal stromal tumor, WT = wild type, ? = not to date

Thus, patients diagnosed with WT pediatric GISTs could either represent an early manifestation of CT or a *forme fruste *of the CT. The time interval from diagnosis of GIST until the appearance of other component/s of the CT averages ≥ 8 years but may be as long as three decades [16,27]. Therefore, these patients should be subjected to regular clinical examination aiming to timely detect possible extraadrenal paraganglioma, pulmonary chondroma and other facultative recently detected components of the triad (leiomyoma of the esophagus and adrenal cortical adenoma).

## Competing interests

The authors declare that they have no competing interests.

Written informed consent was obtained from the patient/the patients parents for publication of this report and any accompanying images. A copy of the written consent is available for review by the Editor-in-Chief of this journal.

## Authors' contributions

All authors read and approved the final manuscript.
